# Receipt of Glucose Testing and Performance of Two US Diabetes Screening Guidelines, 2007–2012

**DOI:** 10.1371/journal.pone.0125249

**Published:** 2015-04-30

**Authors:** Kai McKeever Bullard, Mohammed K. Ali, Giuseppina Imperatore, Linda S. Geiss, Sharon H. Saydah, Jeanine B. Albu, Catherine C. Cowie, Nancy Sohler, Ann Albright, Edward W. Gregg

**Affiliations:** 1 Division of Diabetes Translation, Centers for Disease Control and Prevention, Atlanta, Georgia, United States of America; 2 St. Luke's-Roosevelt Hospital Center, Department of Medicine, Columbia University, New York, New York, United States of America; 3 National Institute of Diabetes and Digestive and Kidney Diseases, National Institutes of Health, Bethesda, Maryland, United States of America; 4 Sophie Davis School of Biomedical Education of The City College of New York, New York, New York, United States of America; Tufts University, UNITED STATES

## Abstract

**Background:**

Screening guidelines are used to help identify prediabetes and diabetes before implementing evidence-based prevention and treatment interventions. We examined screening practices benchmarking against two US guidelines, and the capacity of each guideline to identify dysglycemia.

**Methods:**

Using 2007–2012 National Health and Nutrition Examination Surveys, we analyzed nationally-representative, cross-sectional data from 5,813 fasting non-pregnant adults aged ≥20 years without self-reported diabetes. We examined proportions of adults eligible for diagnostic glucose testing and those who self-reported receiving testing in the past three years, as recommended by the American Diabetes Association (ADA) and the US Preventive Services Task Force (USPSTF-2008) guidelines. For each screening guideline, we also assessed sensitivity, specificity, and positive (PPV) and negative predictive values in identifying dysglycemia (defined as fasting plasma glucose ≥100 mg/dl or hemoglobin A1c ≥5.7%).

**Results:**

In 2007–2012, 73.0% and 23.7% of US adults without diagnosed diabetes met ADA and USPSTF-2008 criteria for screening, respectively; and 91.5% had at least one major risk factor for diabetes. Of those ADA- or USPSTF-eligible adults, about 51% reported being tested within the past three years. Eligible individuals not tested were more likely to be lower educated, poorer, uninsured, or have no usual place of care compared to tested eligible adults. Among adults with ≥1 major risk factor, 45.7% reported being tested, and dysglycemia yields (i.e., PPV) ranged from 45.8% (high-risk ethnicity) to 72.6% (self-reported prediabetes). ADA criteria and having any risk factor were more sensitive than the USPSTF-2008 guideline (88.8–97.7% vs. 31.0%) but less specific (13.5–39.7% vs. 82.1%) in recommending glucose testing, resulting in lower PPVs (47.7–54.4% vs. 58.4%).

**Conclusion:**

Diverging recommendations and variable performance of different guidelines may be impeding national diabetes prevention and treatment efforts. Efforts to align screening recommendations may result in earlier identification of adults at high risk for prediabetes and diabetes.

## Introduction

Diabetes is a leading cause of death, disability, and health care costs in the United States (US) [1,2]. Twenty-nine million Americans have diabetes, though a quarter of them remain unaware of their condition [1]. Additionally, an estimated 86 million Americans have prediabetes [1], a precursor phase in which blood glucose is not yet in the diagnostic range for diabetes, but increases one’s risk of developing type 2 diabetes five- to twelve-fold [3]. Only eleven percent of people with prediabetes are aware of having this condition [4]. Awareness gaps are likely to impede implementation of evidence-based interventions to prevent diabetes [5,6] and its complications [7] among adults with prediabetes and diabetes, respectively.

While the prevalence of diabetes and prediabetes continues to grow in the US [8,9], screening for both of these conditions is hotly debated [10,11]. Diabetes meets many of Wilson and Junger’s criteria for screening [12]: it is a major public health issue; there is an identifiable precursor phase (prediabetes); there are well-accepted glucose testing approaches to identify risk; evidence-based lifestyle and pharmaceutical interventions exist to prevent and control diabetes; and delayed diagnosis leads to progressive organ damage [7,13]. To date, most expert groups have recommended offering glucose testing to individuals deemed to be at high-risk for diabetes [10,14,15]. As such, various expert groups (e.g., the US Preventive Services Task Force [USPSTF] [16], American Diabetes Association [ADA] [17], UK National Institute of Health and Care Excellence [18], among others) have proposed guidelines that recommend whom to test and when. However, little is known about the extent to which these targeted screening recommendations are applied in the U.S. population (i.e., whether those eligible based on guidelines actually receive glucose testing). Secondly, little is known about the performance of these guidelines in identifying diabetes and prediabetes in the United States.

The objectives of this study were: 1) to estimate the proportion of the non-diabetic population that is eligible for diabetes and pre-diabetes screening as recommended by the two most cited US guidelines (USPSTF-2008 and ADA); 2) to estimate the proportion of those eligible for screening who reported receiving glucose testing in the past three years; 3) to examine factors associated with self-reported glucose testing by eligibility status; and 4) to examine the extent to which, by applying the two screening guidelines, dysglycemia is actually identified. Although they have explicitly different goals, we used these two guidelines since USPSTF criteria are commonly used to set health and preventive service coverage levels nationally, and ADA annually provides authoritative diabetes care and prevention recommendations. At the time of submission, USPSTF proposed changes to the screening recommendation, including screening for abnormal blood glucose and type 2 diabetes mellitus in adults with known risk factors for impaired fasting glucose, impaired glucose tolerance, or diabetes [19]. In anticipation of this change to the USPSTF guideline, we also examined self-reported glucose testing patterns by selected major risk factors. These findings have important implications for improving screening practice yields as well as reducing inefficiencies of superfluous testing.

## Methods

### Data source

Data were from the 2007–2012 National Health and Nutrition Examination Surveys (NHANES). NHANES uses a complex, multistage probability sampling design, allowing nationally-representative descriptive statistics of the health status of the non-institutionalized US civilian population [20, 21]. Interview data are collected at households while standardized measurements and biological samples are collected at mobile examination centers. NHANES data are released in 2-year increments. Overall response rates for completing the examination were 75.4%, 77.3%, and 69.5% for the 2007–2008, 2009–2010, and 2011–2012 surveys, respectively.

### Study participants

NHANES adult participants (aged ≥20 years) who had fasted 8-<24 hours were included in the study. We excluded those reporting a previous diagnosis of diabetes (n = 920), pregnant women (n = 70), and participants with missing information (n = 494) regarding diabetes risk factors, receipt of glucose testing, or glycemic measures. The final analytic sample included 5,813 participants.

### Ethics statement

NHANES received approval for human subjects research from the Research Ethics Review Board of the Centers for Disease Control and Prevention’s National Center for Health Statistics. Adult participants provided written informed consent.

### Measurements and case definitions

NHANES survey instruments and protocols have been described extensively [20,21]. Each screening guideline defined people at risk for diabetes, and therefore eligible to receive glucose screening tests, based on a different set of risk factors. We identified variables in the NHANES survey to match these sets of risk factors for dysglycemia (Table 1). For ADA guidelines [17], risk factors included age ≥45 years; body mass index (BMI [calculated as weight in kilograms divided by height in meters squared] ≥25 kg/m^2^); physical inactivity (e.g., no work-, transport-, or recreation-related physical activity in a typical week); family history of diabetes in a first-degree relative; high-risk ethnicity (e.g., non-Hispanic black, Hispanic, and those who did not identify as non-Hispanic white); history of delivering a macrosomic baby or having gestational diabetes; hypertension (average of three blood pressure readings ≥140/90 mmHg, regardless of antihypertensive medication use); dyslipidemia (HDL-cholesterol <35 mg/dL, triglycerides >250 mg/dL, regardless of lipid-lowering medication use); self-reported diagnosis of prediabetes (i.e., impaired fasting glucose, impaired glucose tolerance, or borderline diabetes); and self-reported history of vascular disease (e.g., myocardial infarction, coronary heart disease, or stroke). NHANES did not collect data on history of polycystic ovary syndrome, which is also a risk factor for dysglycemia according to ADA guidelines. For USPSTF-2008 guidelines [16], we defined high-risk as having an average of three blood pressure readings >135/80 mmHg, regardless of antihypertensive medication use.

**Table 1:** Criteria for three screening guidelines and definitions from the National Health and Nutrition Examination Survey, 2007–2012.

ADA^a^ ^,^ ^b^	USPSTF-2008 ^c^
• BMI ≥25 kg/m^2^ [weight in kilograms divided by height in meters squared] plus 1 or more risk factors:	• Average blood pressure from up to three readings >135/80 mm Hg (regardless of antihypertensive medication use)
• Physical inactivity [no work-, transport-, or recreation-related physical activity in a typical week]	
• Family history of diabetes in a first-degree relative	
• High-risk race/ethnicity [non-Hispanic black,—Hispanic, and others who did not identify as non-Hispanic white]	
• History of gestational diabetes or delivering a macrosomic baby	
• Hypertension [average blood pressure ≥140/90 mm Hg]	
• Dyslipidemia [HDL-cholesterol <35 mg/dl or triglycerides >250 mg/dl]	
• History of prediabetes [self-reported prediabetes, impaired fasting glucose, impaired glucose tolerance, or borderline diabetes]	
• History of vascular disease [self-reported myocardial infarction, coronary heart disease, or stroke]	
Or	
• Age ≥45 years	

USPSTF, United States Preventive Services Task Force; ADA, American Diabetes Association; NICE, National Institute for Health and Clinical Excellence; BMI, body mass index.

^a^ Standards of medical care in diabetes—2006. Diabetes Care 2006; 29 Suppl 1: S4–42.

^b^ NHANES did not collect data on polycystic ovary syndrome which is considered a risk factor by ADA guidelines

^c^ Screening for type 2 diabetes mellitus in adults: U.S. Preventive Services Task Force recommendation statement. Annals of Internal Medicine 2008; 148(11): 846–54.

We also identified adults with any major risk factor of: age ≥45 years, body mass index ≥25 kg/m^2^, family history of diabetes, high-risk ethnicity, history of gestational diabetes, blood pressure ≥140/90 mmHg, or self-reported prediabetes. With the exception of self-reported prediabetes, we selected these risk factors because of their inclusion in the proposed new USPSTF guidelines [19]. We included self-reported prediabetes because little is known about patterns of glucose testing and dysglycemia in this growing, high-risk population.

Eligibility for screening at the time of the survey was defined as meeting the criteria for a given guideline. For example, eligibility based on ADA criteria included participants aged ≥45 years or any overweight (BMI ≥25 kg/m^2^) participant aged <45 years with at least one additional risk factor [17]. If an individual did not meet any criterion, then the individual was considered ineligible for glucose testing based on that specific guideline. We defined self-reported receipt of glucose testing as an affirmative answer to the question, “Have you had a blood test for high blood sugar or diabetes within the past three years?”

We defined dysglycemia as prediabetes or undiagnosed diabetes according to blood glucose levels measured in NHANES. Prediabetes was defined as fasting plasma glucose (FPG) 100 to 125 mg/dL or hemoglobin A1c (A1C) 5.7 to <6.5% (39 to <48 mmol/mol). Undiagnosed diabetes was defined as FPG ≥126 mg/dL or A1C ≥6.5% (48 mmol/mol). Any dysglycemia was defined as: A1C ≥5.7% or FPG ≥100 mg/dL [17]. A subgroup of participants also completed an oral glucose tolerance test (OGTT; n = 5,337); therefore, we were able to include 2-hour plasma glucose values (2hrPG) in a second, more conservative, definition of “confirmed” dysglycemia [17,22], defined as meeting all of three criteria: FPG ≥100 mg/dL, 2hrPG ≥140 mg/dL [23], and A1C ≥5.7% (39 mmol/mol).

### Statistical analysis

Our goal was to provide information to clinicians about not only screening practices but the characteristics of adults who reported testing (whether they were actually tested or not) to help them better identify high risk adults. We used SAS, version 9.3 (SAS Institute, Cary, North Carolina) and SAS-callable SUDAAN, version 11 (Research Triangle Park, NC) for statistical analyses, accounting for NHANES’ complex survey design. We estimated the proportion of adults self-reporting receipt of glucose testing in the past three years by eligibility based on each guideline using cross-tabulations (eligible and tested, eligible and not tested, ineligible and tested, ineligible and not tested). We then compared factors associated with glucose testing among mutually exclusive groups according to eligibility status based on current US guidelines (i.e., USPSTF-2008 and ADA). Using logistic regression modeling, we also calculated crude prevalence estimates for self-reported glucose testing by major risk factors and prevalence ratios, adjusted for age, sex, race/ethnicity, education, income, insurance status, and usual place of care. Finally, we assessed the performance (sensitivity, specificity, positive predictive value [PPV], and negative predictive value [NPV]) of the screening guidelines to identify dysglycemia and undiagnosed diabetes using blood glucose levels measured in the NHANES as the gold standard.

## Results

**Fig 1:**
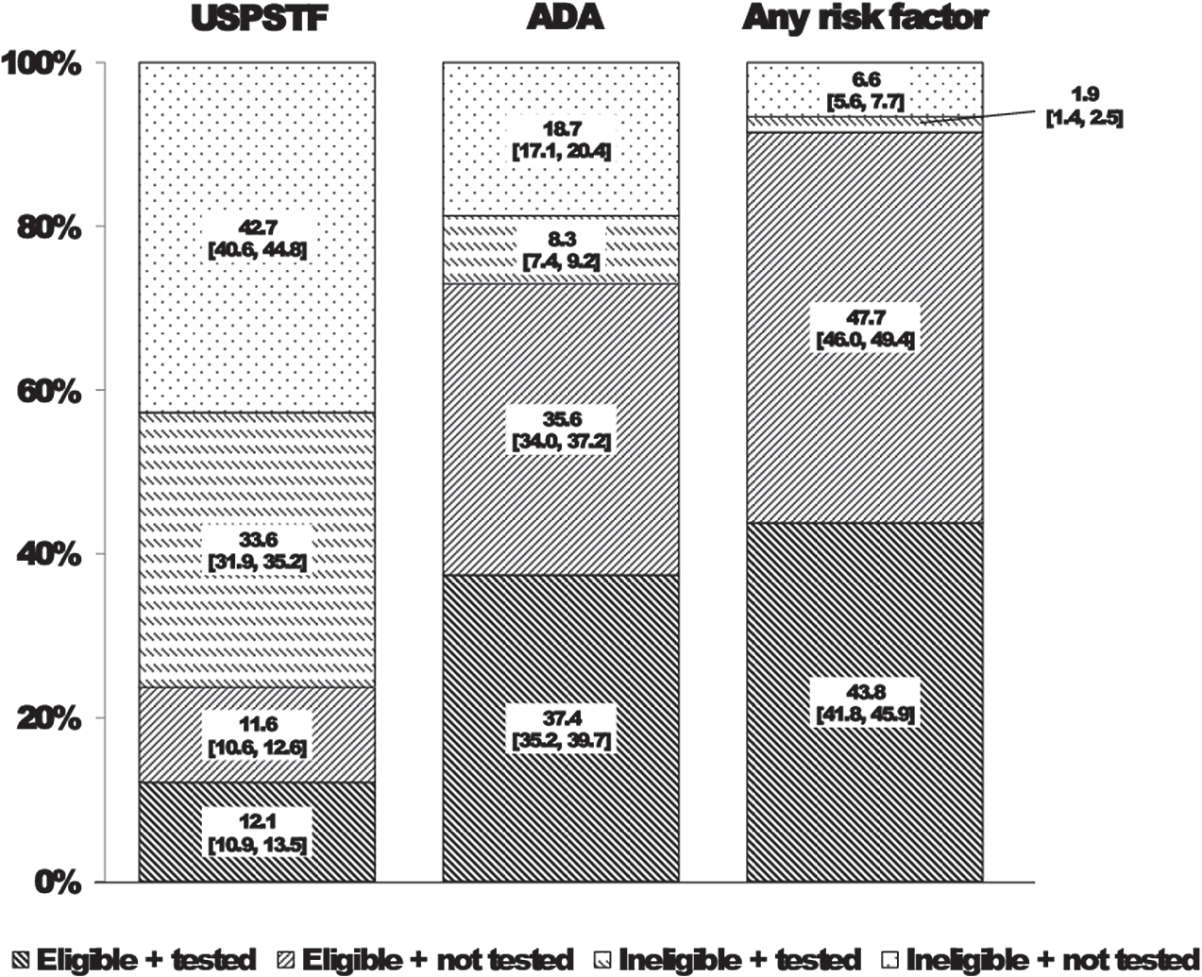
Percentage of US adults reporting receipt of glucose testing by eligibility status according to diabetes screening guidelines. Abbreviations: USPSTF, United States Preventive Services Task Force; ADA, American Diabetes Association. Data were from 5,813 adults without diagnosed diabetes in the 2007–2012 National Health and Nutrition Examination Survey. Receipt of glucose testing was defined as an affirmative answer to the question, “Have you had a blood test for high blood sugar or diabetes within the past three years?” Any risk factor is defined as: age ≥45 years, body mass index ≥ 25 kg/m2, family history of diabetes, high-risk ethnicity, history of gestational diabetes or prediabetes, or blood pressure ≥140/90 mmHg.

In 2007–2012, 23.7% and 73.0% of US adults without diagnosed diabetes met USPSTF-2008 and ADA eligibility for glucose testing, respectively; while 91.5% of adults had at least one major diabetes risk factor (Fig 1). Extrapolating to the 2013 Census population aged ≥20 years without diagnosed diabetes (approximately 213,887,500), these percentages represent roughly 50.8 million, 156.2 million, and 195.7 million adults at risk for diabetes, respectively. Of those USPSTF- or ADA-eligible, 50.7% [48.4–52.9] reported receiving glucose testing within the past three years. During the 6-year period, estimates of self-reported glucose testing significantly increased among ADA-eligible (47.0% [45.0–48.9] in 2007–2008 to 55.4% [51.1–59.6] in 2011–2012; *P* = 0.005) but not USPSTF-eligible adults (48.5% [43.7–53.3] to 54.8% [49.3–60.2]; *P* = 0.24) (data not shown). Greater proportions of eligible adults aged ≥45 years compared to their younger eligible counterparts aged <45 years reported receiving glucose testing within the past three years: 55.1% vs 42.4% (USPSTF-2008) and 55.6% vs 41.3% (ADA); both *P*-values<0.01 (data not shown). Based on the USPSTF-2008 guidelines, 33.6% of US adults were ineligible but still reported receiving testing. Similarly, 8.3% of adults reported being tested and were classified as ineligible by ADA criteria; while only 1.9% of the population had no major risk factors but reported being tested (Fig 1).

Examining the commonly-used US practice guidelines, about 21% of non-diabetic US adults were eligible for glucose testing based on both ADA and USPSTF-2008 guidelines, while almost 52% were eligible based on ADA criteria alone and 25% were ineligible on both ADA and USPSTF-2008 guidelines. Only about 2% were USPSTF-eligible, but not ADA-eligible. (data not shown).

Table 2 shows sociodemographic and clinical factors associated with glucose testing according to ADA and USPSTF-2008 screening eligibility. Non-diabetic U.S. adults who were classified as eligible for glucose testing based on both guidelines and were tested tended to be older, have higher mean BMI, or have larger waist circumferences, compared with those eligible but not tested (*P*-values <0.001). For adults only eligible based on ADA and not USPSTF-2008 guidelines, those tested tended to be older or more likely to be female or more educated than those not tested (*P*-values <0.01). Of those that were ADA- and USPSTF-eligible or just ADA-eligible, those not receiving glucose testing were more likely to be younger, poorer, uninsured, or have no usual place of care compared to those receiving testing (*P*-values <0.05). Even among those that were ineligible by both guidelines, a higher proportion of those not tested were uninsured (*P* = 0.001) or had no usual place of care compared to those tested (*P* = 0.01). Compared to non-diabetic U.S. adults that were ADA-eligible for glucose testing, higher proportions of combined USPSTF- and ADA-eligible adults had dysglycemia (range: 55.6–67.7% vs. 44.6–57.8% [ADA only]). Of the group that both guidelines classified as ineligible, 16.9–18.6% had prediabetes and ≤0.2% had undiagnosed diabetes. Additionally, among those eligible by either ADA or USPSTF-2008 criteria, receipt of glucose testing was independently associated with age, education, insurance coverage, usual place of care, and BMI, even after controlling for sociodemographic and clinical characteristics (data not shown).

**Table 2:** Characteristics of adults without diagnosed diabetes according to US guideline eligibility groups^a^ who did and did not report receipt of glucose testing^b^ in past 3 years.

	Total		Eligible based on both ADA and USPSTF		Eligible based on ADA, but not USPSTF		Ineligible based on both ADA and USPSTF	
			*Currently recommended for screening*		*Potentially identifiable with new changes in 2015*		*Not recommended for screening*	
	*Not Tested*	*Tested*	*Not Tested*	*Tested*	*Not Tested*	*Tested*	*Not Tested*	*Tested*
Unweighted n	3040	2773	682	783	1515	1613	770	342
Population size (millions)	99.6	83.8	18.3	20.8	47.0	47.9	31.3	13.7
Age (years), % (SE)								
20–44 years	58.6 (1.4)	38.3 (1.6)**	26.4 (1.9	20.6 (2.1)**	41.0 (2.1)	26.5 (1.6)**	100.0	100.0
45–64 years	31.5 (1.3)	40.9 (1.5)	48.7 (2.1)	48.3 (2.2)	47.8 (2.1)	50.6 (1.7)	n/a	n/a
≥65 years	9.9 (0.5)	20.8 (0.8)	25.0 (1.9)	31.1 (1.9)	11.3 (0.9)	22.9 (1.0)	n/a	n/a
Age (years), mean (SE)	42.1 (0.4)	50.2 (0.5)**	53.9 (0.6)	57.3 (0.6)**	46.1 (0.6)	52.7 (0.5)**	30.0 (0.3)	32.1 (0.5)**
Male, % (SE)	51.8 (1.0)	44.6 (1.1)**	54.7 (2.3)	49.9 (1.9)	50.9 (1.4)	43.0 (1.7)**	49.4 (2.1)	39.7 (3.3)*
Race/ethnicity, % (SE) ^c^								
NH white	68.0 (2.0)	70.1 (2.2)**	63.8 (3.3)	68.7 (3.0)	63.8 (2.5)	69.3 (2.4)**	76.2 (1.9)	75.2 (2.9)
NH black	9.9 (1.0)	11.7 (1.1)	15.1 (1.7)	15.5 (2.0)	10.1 (1.1)	11.8 (1.2)	6.7 (0.9)	5.0 (0.9)
Hispanic	14.8 (1.5)	12.3 (1.4)	13.3 (1.9)	10.9 (1.5)	19.6 (2.0)	13.8 (1.6)	9.3 (1.3)	9.9 (1.4)
Education <HS, % (SE)	18.1 (1.1)	16.4 (1.1)	21.5 (2.3)	18.8 (1.8)	21.0 (1.5)	16.5 (1.3)**	11.7 (1.6)	13.4 (2.3)
At or below poverty, % (SE)	16.7 (1.2)	12.3 (0.9)**	18.6 (2.1)	12.1 (1.5)**	16.0 (1.1)	11.9 (1.1)**	16.4 (1.6)	14.4 (2.1)
Uninsured, % (SE)	27.4 (1.1)	12.9 (0.8)**	23.8 (1.8)	13.4 (1.5)**	27.2 (1.5)	11.4 (1.1)**	29.3 (2.3)	17.9 (2.4)**
No usual place of care, % (SE)	21.3 (1.0)	7.3 (0.6)**	19.1 (1.7)	6.1 (1.0)**	21.1 (1.3)	5.6 (0.7)**	22.4 (2.0)	14.4 (2.0)*
Physical inactivity, % (SE)	18.8 (1.1)	21.0 (1.0)	24.9 (2.0)	26.3 (1.7)	23.9 (1.6)	23.3 (1.7)	8.5 (1.1)	6.7 (1.0)
BMI (kg/m^2^), mean (SE)	27.3 (0.1)	29.3 (0.2)**	29.2 (0.3)	31.3 (0.4)**	28.8 (0.2)	30.0 (0.2)**	23.9 (0.2)	24.4 (0.3)
Waist circumference (cm), mean (SE)	94.5 (0.4)	100.3 (0.4)**	101.0 (0.6)	106.0 (0.7)**	98.6 (0.5)	102.0 (0.5)**	84.8 (0.5)	86.7 (1.0)*
SBP (mmHg), mean (SE)	118.4 (0.4)	120.7 (0.4)**	138.3 (0.8)	140.3 (0.8)	115.2 (0.4)	115.3 (0.3)	110.8 (0.5)	109.4 (0.6)
DBP (mmHg), mean (SE)	69.8 (0.4)	70.2 (0.4)	80.6 (0.6)	79.3 (0.7)	67.5 (0.3)	67.0 (0.3)	65.5 (0.5)	66.0 (0.6)
FPG (mg/dL), mean (SE)	95.9 (0.3)	99.5 (0.4)**	101.4 (0.8)	103.6 (1.0)	97.4 (0.5)	100.3 (0.5)**	90.6 (0.4)	91.3 (0.6)
A1c (%), mean (SE)	5.4 (0.01)	5.5 (0.01)**	5.6 (0.03)	5.7 (0.03)*	5.5 (0.02)	5.6 (0.01)**	5.2 (0.01)	5.2 (0.02)
PreDM, % (SE)	35.2 (1.1)	48.0 (1.3)**	49.6 (2.7)	58.8 (3.1)*	42.4 (1.8)	52.1 (1.4)**	16.9 (1.6)	18.6 (2.5)
DM, % (SE)	2.2 (0.3)	5.4 (0.5)**	6.0 (0.9)	8.5 (1.3)	2.2 (0.4)	5.7 (0.7)**	0.1 (0.1) ^d^	0.2 (0.2) ^d^

N = 5813, representing 183 million adults without diagnosed diabetes

Abbreviations: USPSTF, United States Preventive Services Task Force; ADA, American Diabetes Association; HS, high school; BMI, body mass index; FPG, fasting plasma glucose; SBP, systolic blood pressure; DBP, diastolic blood pressure; preDM, prediabetes defined by FPG 100–125 mg/dL or A1C 5.7–6.4%; DM, diabetes defined by FPG ≥126 mg/dL or A1C ≥6.5%.

^a^ Insufficient sample size for participants eligible for screening based on USPSTF but not ADA criteria (n = 108, representing 4.4 million adults without diagnosed diabetes).

^b^ Defined as an affirmative answer to the question, “Have you had a blood test for high blood sugar or diabetes within the past three years?”

^c^ Participants of other race/ethnicity included in analysis, but results not shown

^d^ Unstable estimate due to insufficient sample size or relative standard error greater than 30%.

* P-value <0.05;

**P-value <0.01 for comparing tested and not tested, calculated from Wald *F* test or *t* test.

Table 3 shows crude prevalence estimates and adjusted prevalence ratios for self-reported glucose testing and dysglycemia according to major risk factor subgroups. Overall, compared to non-diabetic adults without any major risk factors, those with at least one were 63% more likely to report receiving a glucose test in the past 3 years and 93% more likely to have dysglycemia (*P*-values<0.001). Glucose testing estimates were highest among non-diabetic adults with the following risk factors: previous diagnosis of prediabetes (80.3%), gestational diabetes (57.1%), age ≥45 years (55.6%), followed by high blood pressure (54.4%). Prevalence of glucose testing ranged 44.0–50.2% for non-diabetic overweight adults, those with a family history of diabetes, or those of high-risk ethnicity. Each risk factor was significantly associated with dysglycemia, independent of sociodemographic characteristics. Those with individual risk factors had 12 to 117% greater prevalence of dysglycemia compared to those without risk factors; while, dysglycemia prevalence exceeded 60% only for those aged ≥45 years, those with high blood pressure, and those with a history of prediabetes.

**Table 3:** Prevalence of self-reported receipt of glucose testing in past 3 years and dysglycemia according to selected major risk factors among US adults without diagnosed diabetes.

	Represented Population^a^	Self-reported glucose testing^b^		Dysglycemia^c^	
		Crude prevalence	Adjusted prevalence ratio^d^	Crude prevalence	Adjusted prevalence ratio^d^
	n, millions	% (SE)	PR (95% CI)	% (SE)	PR (95% CI)
**Total**	183.4	45.7 (1.0)		44.7 (1.0)	
**Age**					
20–44 y (reference)	90.5	35.5 (1.2)	1.00	28.5 (1.3)	1.00
≥45 y	92.9	55.6 (1.3)	1.44 (1.31, 1.57)	60.5 (1.2)	2.17 (1.96, 2.41)
**Overweight or obese**					
<25 kg/m^2^ (reference)	61.2	36.8 (1.6)	1.00	31.7 (1.7)	1.00
≥25 kg/m^2^	121.0	50.2 (1.0)	1.32 (1.21, 1.44)	51.3 (1.1)	1.45 (1.30, 1.63)
**Family history of diabetes**					
No (reference)	122.4	43.6 (1.3)	1.00	41.7 (0.9)	1.00
Yes	61.0	49.9 (1.5)	1.12 (1.03, 1.21)	50.7 (1.8)	1.18 (1.09, 1.28)
**High-risk ethnicity**					
No (reference)	126.5	46.5 (1.2)	1.00	44.2 (1.3)	1.00
Yes	56.9	44.0 (1.3)	1.12 (1.05, 1.20)	45.8 (1.3)	1.12 (1.03, 1.21)
**History of gestational diabetes**					
No (reference)	169.1	44.7 (1.0)	1.00	44.2 (1.0)	1.00
Yes	14.3	57.1 (2.9)	1.15 (1.02, 1.29)	50.3 (3.2)	1.19 (1.05, 1.34)
**Hypertension**					
No (reference)	161.6	44.5 (1.1)	1.00	41.8 (1.1)	1.00
Yes	21.8	54.4 (2.0)	1.11 (1.01, 1.22)	66.4 (2.2)	1.27 (1.15, 1.40)
**History of prediabetes**					
No (reference)	174.7	44.0 (1.0)	1.00	43.3 (1.0)	1.00
Yes	8.7	80.3 (2.7)	1.69 (1.54, 1.84)	72.6 (3.3)	1.55 (1.37, 1.76)
**Any risk factor** ^e^					
No (reference)	15.5	22.2 (2.8)	1.00	12.1 (2.4)	1.00
Yes	167.9	47.9 (1.0)	1.63 (1.31, 2.03)	47.7 (1.0)	1.93 (1.33, 2.82)

Abbreviations: SE, standard error; PR, prevalence ratio.

^a^ Population sizes may not sum to 183 million due to rounding

^b^ Defined as an affirmative answer to the question, “Have you had a blood test for high blood sugar or diabetes within the past three years?”

^c^ Defined as fasting plasma glucose ≥100 mg/dL or A1c ≥5.7%

^d^ Estimated from logistic regression models, adjusted for age, sex, race/ethnicity, education, income, insurance status, and usual place of care

^e^ Defined as any of: age ≥45 years, body mass index ≥25 kg/m^2^, family history of diabetes, high-risk ethnicity, history of gestational diabetes, blood pressure ≥140/90 mmHg, or self-reported prediabetes.

Regarding performance of the guidelines, Table 4 provides estimates of sensitivity, specificity, PPV, and NPV (and respective 95% CI’s) for each of the three screening guidelines using NHANES laboratory tests as the standard for identifying any dysglycemia,”confirmed” dysglycemia, and undiagnosed diabetes. Compared with USPSTF-2008 guidelines, ADA criteria were more sensitive in identifying those with any dysglycemia (Percentage of true positives over all positives for USPSTF-2008: 31.0% [29.0, 33.1] vs. ADA: 88.8% [87.0, 90.4]) but less specific (Percentage of true negatives over all negatives: 82.1% [79.7, 84.3] vs. 39.7% [36.8, 42.7]). Restricting to an increasingly more conservative definition of dysglycemia, both guidelines yielded better sensitivity and declining specificity. Using any major risk factor to identify dysglycemia yielded the highest sensitivity and lowest specificity compared to ADA and USPSTF-2008 guidelines.

**Table 4:** Performance^a^ of screening criteria in identifying dysglycemia among US adults without diagnosed diabetes.

Dysglycemia definition	Guideline	Sensitivity ^b^	Specificity ^c^	PPV ^d^	NPV ^e^
FPG ≥100 mg/dL orA1c ≥5.7% ^f^	USPSTF-2008	31.0 (29.0, 33.1)	82.1 (79.7, 84.3)	58.4 (54.5, 62.1)	59.6 (57.5, 61.7)
	ADA	88.8 (87.0, 90.4)	39.7 (36.8, 42.7)	54.4 (52.1, 56.5)	81.5 (78.6, 84.1)
	Any major risk factor ^g^	97.7 (96.4, 98.5)	13.5 (11.7, 15.4)	47.7 (45.7, 49.8)	87.9 (82.3, 91.9)
All 3 of: FPG ≥100 mg/dL, A1c ≥5.7% ^f^, and 2hrPG ≥140 mg/dL ^h^	USPSTF-2008	40.6 (36.1, 45.3)	78.0 (76.4, 79.6)	13.9 (11.7, 16.4)	93.8 (92.8, 94.6)
	ADA	98.3 (95.9, 99.3)	28.9 (26.7, 31.3)	10.8 (9.6, 12.1)	99.5 (98.7, 99.8)
	Any major risk factor ^g^	99.8 (98.7, 100.0)	8.8 (7.7, 10.1)	8.7 (7.7, 9.8)	99.8 (98.7, 100.0)
Undiagnosed DM (FPG ≥126 mg/dL or A1c ≥6.5% ^f^)	USPSTF-2008	42.9 (35.0, 51.1)	77.0 (75.3, 78.6)	6.6 (5.3, 8.2)	97.3 (96.7, 97.7)
	ADA	98.7 (97.3, 99.4)	27.9 (25.9, 30.1)	4.9 (4.3, 5.7)	99.8 (99.6, 99.9)
	Any major risk factor ^g^	99.4 (97.3, 99.9)	8.8 (7.6, 10.1)	4.0 (3.5, 4.6)	99.7 (98.8, 99.9)

Abbreviations: PPV, positive predictive value; NPV, negative predictive value; USPSTF, United States Preventive Services Task Force; ADA, American Diabetes Association; DM, diabetes mellitus; FPG, fasting plasma glucose; 2hrPG, 2-hr plasma glucose from an oral glucose tolerance test.

^a^ Data are presented as weighted percentages and 95% confidence intervals based on data from 5,813 adults without diagnosed diabetes, representing 183 million

^b^ Probability of being eligible for screening among those with dysglycemia (true positives)

^c^ Probability of being ineligible for screening among those without dysglycemia (true negatives)

^d^ Probability of having dysglycemia among those eligible for screening

^e^ Probability of not having dysglycemia among those ineligible for screening

^f^ A1c values 5.7% and 6.5% correspond to A1c values 39 mmol/mol and 48 mmol/mol.

^g^ Defined as any of: age ≥45 years, body mass index ≥25 kg/m^2^, family history of diabetes, high-risk ethnicity, history of gestational diabetes, blood pressure ≥140/90 mmHg, or self-reported prediabetes

^h^ Subpopulation of adults with available 2-hour PG values; N = 5,337.

Among adults that the USPSTF-2008 guidelines recommended for glucose testing, 58.4% (54.5, 62.1) actually had any dysglycemia, while this PPV was 54.4% (52.1, 56.4) and 47.7% (45.7, 49.8) for the ADA criteria and any major risk factor, respectively. Among those with “confirmed” dysglycemia or undiagnosed diabetes, the PPV of both guidelines diminished considerably, ranging from 4.9% to 13.9%. However, those that the guidelines identified as ineligible for glucose testing, the proportion without “confirmed” dysglycemia or undiagnosed diabetes (or the NPV) neared 100%.

## Discussion

Amidst the large and growing national diabetes burden and intense debates regarding how to improve detection, our study is the first national-level assessment—of which we are aware—that both compares screening guideline performance and examines receipt of glucose testing by eligibility and major factor status. Our findings show wide variation across US expert guidelines in terms of proportions of US adults eligible for glucose testing, as well as wide variation in the yield of testing when guidelines are applied (i.e. PPV). Even so, and importantly, gaps exist in testing practices as only 50–60% of US adults who met any of the glucose testing criteria reported receiving testing (i.e. appropriate testing).

Among those eligible by either US guideline or just ADA guidelines, individuals not receiving glucose testing were more likely to have low education, low household income, and to lack health insurance or a usual health care provider than individuals who received testing. These factors may also be interconnected, as health insurance is linked to the likelihood of having a usual care provider. As a comparison, a study examining electronic medical records of 46,991 persons attending a large academic physician practice during 2005–2007 showed that 72.0% met either USPSTF or ADA criteria for screening, and 85.3% of them received glucose testing [24]. This same analysis also showed a sizeable difference in glucose testing uptake between uninsured (54.9%) and insured (85.4%). It will be important to continue to evaluate glucose testing practices as access to preventive services changes, especially for lower socioeconomic groups.

It is expected that future USPSTF-approved preventive services will be provided without cost-sharing [25] which elevates the importance of this particular guideline’s predictive validity for identifying dysglycemia. As demonstrated in our data and previous reports [24,26], USPSTF-2008 criteria are the least sensitive, resulting in glucose testing in only half of all dysglycemia cases. This may be in part because USPSTF criteria were developed to identify undiagnosed diabetes. In 2014, USPSTF proposed screening for abnormal blood glucose and type 2 diabetes mellitus in adults who are at increased risk for diabetes although the final, specific risk factor criteria were yet to be published at the time of this submission [19]. This change better aligns with the screening criteria of the ADA and organizations that endorse using risk assessment tools to identify adults at high risk. Applying the 2008 USPSTF guideline to the 2013 Census adult population without diagnosed diabetes would identify at least 50 million adults; while proposed changes could potentially identify 91.5% of the non-diabetic population, or 195 million adults, eligible for glucose testing.

Earlier detection is important. In the Diabetes Prevention Program Outcomes Study, providing intensive lifestyle modification to the intervention group three years earlier than the control group continues to have incremental benefits in terms of cumulative incidence of diabetes, need for medications, as well as hypertension and lipid control [5]. In addition, those achieving improved beta-cell function and even transient regression to normal glucose levels experienced lower conversion to diabetes [27]. Lastly, 10–20% of persons newly diagnosed with diabetes have retinopathy [28] and national surveillance shows that 41.7% of those with undiagnosed diabetes and 17.7% of people with prediabetes have chronic kidney disease [29]. Almost half of all undiagnosed cases of diabetes have hypertension, lipid abnormalities, or cardiovascular and chronic kidney diseases [26]. Delayed diagnosis hinders the possibility of earlier intervention to slow the disease processes of dysglycemia and its co-morbidities.

On the other hand, as a result of the ADA criteria’s lower thresholds for recommending testing, approximately three-quarters of the United States’ entire non-diabetic adult population (~200 million people) would become eligible for glucose testing. Though our data show that this would identify over 90% of dysglycemia of any form, there would be higher costs and inefficiencies (i.e. low yield) associated with this population-wide screening approach. There is an ongoing debate regarding cost-effectiveness of universal screening versus offering glucose testing to only high-risk individuals. With universal testing, likelihood of low yield (perhaps due to low prevalence in the population) and subsequent over-testing and excess costs are concerns, especially for those younger than 35 years old [15]. With targeted testing, an extra step of risk assessment is needed, and since current risk assessment guidelines recommend varied thresholds within (e.g., USPSTF-2008 and ADA) and across populations (e.g., there are European, Indian, and other race/ethnicity group risk scores) at which a glucose test should be offered, the balance between missed cases, over-testing, and costs becomes blurred and unclear. Others have raised concerns about collateral harms of such widespread testing, including physical discomfort (e.g., pain) and potentially psychosocial harms like increased anxiety [30] or discrimination, although recent studies suggest that psychological impacts are minimal and diminish with time [10,31].

Since resources are required to care for the added burden of cases that are newly detected through screening, some guidelines also consider cost-effectiveness data. The cost-effectiveness of screening for dysglycemia depends both on the yield in the population as well as the cost-effectiveness of interventions to address prediabetes or diabetes [32,33]. Structured lifestyle modification and metformin are highly cost-effective interventions for people with prediabetes [34]. Additionally, long-term data regarding more intensive treatment in 5,102 persons with newly-diagnosed diabetes in the United Kingdom Prospective Diabetes Study showed reduced micro- and macro-vascular complications and deaths [7]. On the basis of these data, modeling studies suggest screening for both prediabetes and diabetes together (dysglycemia) followed by lifestyle or pharmacotherapies is more cost-effective than screening for just diabetes [10,14]. We therefore used dysglycemia as the outcome to evaluate screening guideline performance in this study. Even so, our data show that the more inclusive ADA recommendation has low specificity and PPV, which would likely result in misused resources if greater proportions of those eligible were to actually receive testing.

Our study is subject to a few limitations. We considered unidentified individuals with diabetes and prediabetes based on elevated FPG or A1C values only. Since we chose not to include OGTT data in all analyses, the prevalence of dysglycemia might be underestimated in our study population. Adults with previously diagnosed prediabetes were included in the analysis, as this is one of ADA’s criteria. Excluding these individuals had little influence on our findings. Furthermore, our estimates are based on cross-sectional measures which limit inferences of true, longitudinal predictive validity. Also, prevalence estimates for self-reported glucose testing may be subject to some uncertainty. Receipt of glucose testing may be underreported due to recall bias and glucose tests conducted, either singly or as part of a blood test panel, without the patient’s knowledge. Prevalence may also be overestimated and could vary according to patient characteristics. For example, individuals with a family history of diabetes may be more aware of their risk and therefore more likely to report testing whether it occurred or not. Similarly, women with a history of GDM and older adults who have more engagement with the health care system may over-report their glucose testing history. However, despite the potential errors in reporting, on a national scale, self-report remains the only viable method of measuring receipt of glucose testing as claims data currently do not cover a representative sample of the whole country. Second, self-reported glucose testing remains an important indicator to monitor over time in light of the growing diabetes epidemic and evolving recommendations for screening adults at high risk for diabetes. In addition, we have no information on the type of glucose testing received (e.g., random versus fasting versus OGTT), though there is variation in performance across tests. There may also be misclassification of glycemic status and characteristics due to the gap between the time when participants received glucose testing and when they were surveyed. Lastly, revisions to the USPSTF guideline were being considered but had not been finalized at the time of publication. Preliminary communication [19] suggests that the new USPSTF guidelines will encourage testing for persons with any major diabetes risk factor (i.e., age, family history, BMI, etc.) and therein be more aligned with ADA guidelines than the prior USPSTF guidelines. Although these recommendations have not been finalized, one of our goals was to compare the performance of both USPSTF guidelines (2008 recommendation *vs* the newer “any major risk factor” recommendation) to examine the potential impact of changes to this guideline on the population at risk as well as patterns of glucose testing by risk factor status.

Despite these limitations, our data are nationally-representative, recent, and the substantial sample size provides stable robust estimates. We evaluated two US guidelines and had variables for all of the most common diabetes risk factors. Additionally, screening guideline performance was comprehensively examined using sensitivity, specificity, PPV, and NPV measures.

### Conclusions

Prediabetes and diabetes prevalence continue to grow in the United States. Primary prevention programs are being initiated across states, delivering evidence-based lifestyle modification programs [35]. Health reforms are underway which may improve access and transform healthcare from a fee-for-service to fee-for-quality paradigm, which, in turn, may impact current gaps in diabetes care [36]. However, all of these efforts are predicated on accurately identifying people who will benefit. As such, detection of dysglycemia is an inseparable component of diabetes prevention and care. Which screening guideline to follow and the optimal sensitivity, specificity, or both required depends on one’s goals and the availability and accessibility of resources. Our data show important gaps and substantial variation in guideline-recommended eligibility for screening and performance of these tools. This divergence is important and may promote indecisiveness and confusion among care providers. As such, to narrow the gaps and take advantage of evolving policies and practices, future steps might include multi-stage screening emphasizing risk scoring approaches and decision analysis. Until then, better utilization of glucose testing guidelines is needed.
